# The cell sets the tone

**DOI:** 10.7554/eLife.37888

**Published:** 2018-06-06

**Authors:** Jonna Alanko, Michael Sixt

**Affiliations:** 1 Institute of Science and Technology AustriaKlosterneuburgAustria

**Keywords:** chemokine signalling, embryo development, weak regulatory linkage, signalling bias, chemokine, ligand bias, Zebrafish

## Abstract

In zebrafish larvae, it is the cell type that determines how the cell responds to a chemokine signal.

**Related research article** Malhotra D, Shin J, Solnica-Krezel L, Raz R. 2018. Spatio-temporal regulation of concurrent developmental processes by generic signaling downstream of chemokine receptors. *eLife* **7**:e33574. doi: 10.7554/eLife.33574

In the body, cells constantly ‘talk’ to each other via extracellular signaling molecules. When one of these molecules binds to a receptor on a cell, it generally activates a signaling pathway that leads to this cell doing something.

Chemokines are signaling molecules that mainly direct how cells migrate during immunity and developmental processes. Vertebrates have more than 40 chemokines and 20 chemokine receptors, which all belong to the well-known class of G protein coupled receptors. There is a certain amount of redundancy in the system, with some chemokines being able to bind to more than one type of receptor, and some receptors being able to bind different types of chemokines (Rot and von Andrian, 2004). Chemokines likely evolved to orchestrate the trafficking of numerous different types of immune cells, but some are also important in embryonic development (Wang and Knaut, 2014). Given the number of different signaling molecules and receptors involved, and the range of roles they play, how does the chemokine receptor-ligand system encode specificity? In other words, how does a cell ‘understand’ which pathway to activate when a chemokine binds to a chemokine receptor on its surface?

To answer this, researchers concentrated on the differences between the signaling pathways triggered by different chemokine receptors. They found that there is a ‘ligand bias’: a receptor can trigger different cellular responses depending on the ligand it binds (Steen et al., 2014). In these cases, the cell ‘knows’ how to respond to a signal because a specific ligand-receptor combination activates a unique cellular pathway (Figure 1A). Now, in eLife, Erez Raz of the University of Münster and colleagues – including Divyanshu Malhotra (Münster), Jimann Shin and Lilianna Solnica-Krezel (both at Washington University School of Medicine) – report results of experiments in zebrafish larvae that challenge the importance of specificity in chemokine recognition (Malhotra et al., 2018).

**Figure 1.**
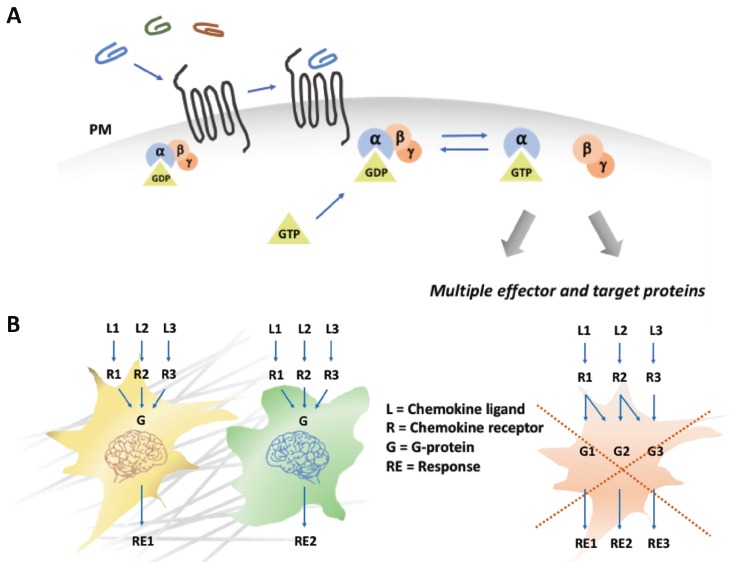
Chemokines trigger a 'yes or no' response in cells. (**A**) When a chemokine (blue, green or brown line) binds to a chemokine receptor (black lines) embedded in the plasma membrane (PM; grey) of a cell, a G protein formed of three subunits (α, ß, γ), one of which (α) is attached to a molecule called GDP, is recruited. The GDP is then replaced with a molecule known as GTP, the G protein dissociates, and the different subunits go on to activate a range of different cellular pathways. (**B**) Different models can explain how chemokines signal within a cell. In the first model (left), different chemokines (L1, L2, L3) bind to their corresponding receptors (R1, R2, R3) and activate a generic G protein mediated pathway (**G**) in two types of cells (in yellow and green). The final response (RE1, RE2) triggered by a chemokine is ultimately dependent on interpretation modules (depicted as cellular brains) that are specific to the cell type, rather than on the identity of the signaling chemokine-receptor complex. The second model (right) proposes that each chemokine-receptor pairs activates a specific cellular pathway that determines a particular cellular response. The work by Malhotra et al. supports the first model (Malhotra et al., 2018).

The researchers took advantage of the fact that zebrafish have a duplicated genome, which means that many of the receptors and ligands come in two versions. Malhotra et al. also made use of the fact that chemokines act in a range of distinct developmental phenomena, such as the migration of germ cells, the adhesion of endoderm cells and the specification of cell fate during gastrulation. They used a combination of genetic approaches, together with imaging-based readouts, to test if receptor-ligand interactions (and the resulting signals) specified the biological process, or if the response depended on the cell type. In their genetic manipulations, the researchers gradually went from subtle to radical. They started by swapping the receptor-ligand pairs that arose due to the duplication of the zebrafish genome. The most extreme changes involved replacing a receptor-ligand pair with a pair that was involved in a completely different developmental process.

From all these experiments, Malhotra et al. got a surprisingly clear answer to their question. The cells more or less always behaved the way they would have if they still had their original receptors: germ cells migrated (albeit to the wrong place), endoderm cells adhered, and they differentiated during gastrulation. While most chemokines signal through the cell via two families of G protein subunits, Gαi and G12/13, further manipulations confirmed that all the different receptors used in the study signaled through the Gαi pathway. This means that the role of the chemokine receptors is to switch on the Gαi signaling cascade; it is then up to the cell to interpret that ‘yes or no’ signal and to trigger the right molecular processes. It is the cell type that dictates the response, not the receptor-ligand pair (Figure 1B). In simple words: no matter which chemokine a specific cell type 'smells', it will always respond the way it 'wants'. This is surprising given the wealth of research that describes different signal modalities for different chemokine receptors.

However, a large number of signaling processes, including many that involve G proteins, also work in such a modular way. A typical example is the system used by vertebrates to discriminate between different odors. In mice, over a thousand different G protein-coupled odor receptors are expressed in the tissue that lines the nose. Yet, a single olfactory sensory neuron does not carry a thousand different receptors, each signaling via a specific pathway. Instead, each neuron only expresses one type of receptor, which responds to only one chemical, and these neurons all work together to identify odors (Buck, 2000). An even more extreme case is the immune system, where every clone of B or T cells carries an individually assembled and therefore specific receptor at its surface. However, all these receptors signal to the cell via the same conserved pathway (Nussenzweig, 1998).

Such funneling of information through one common pathway creates a ‘weak regulatory linkage’ (Gerhart and Kirschner, 2007). In this situation, the input (cell senses something) is coupled to the output (cell does something) via a single conserved process that does not convey any specific information: rather it just signals yes or no. This process can be a membrane potential, a flux of calcium ions or a Gαi signal. This means that inputs and outputs, such as regulatory signals and functional responses, are free to evolve independently from each other. The work of Malhotra et al. provides an excellent example of weak regulatory linkage in the chemokine system.
